# Supplementation of bee pollen in high concentrate diets enhances the health status and production performance of goats

**DOI:** 10.3389/fvets.2026.1780550

**Published:** 2026-04-22

**Authors:** Zehao Tan, Lizhi Wang, Zhisheng Wang, Bai Xue, Quanhui Peng, Rui Hu, Jianxin Xiao

**Affiliations:** Animal Nutrition Institute, Sichuan Agricultural University, Chengdu, Sichuan, China

**Keywords:** bee pollen, goat, growth performance, high concentrate diets, immunity, rumen fermentation

## Abstract

This study investigated the effects of supplementing bee pollen (BP) in high-concentrate diets (HCD) on the health status and production performance of goats. Twenty Jintang Black goats, similar age and weighing 33.36 ± 1.34 kg, were randomly assigned to the CON group (basal diet) and the BP group (basal diet + 0.5% BP) for a 30-day feeding trial. Results indicated that BP supplementation significantly increased average daily gain by 36.55% (*p* < 0.05), and decreased Feed-to-Gain ratio (*p* < 0.05) of goats. Apparent digestibility of dry matter, crude protein, and neutral detergent fiber was also significantly improved (*p* < 0.05). Regarding rumen fermentation parameters, BP significantly increased valeric acid concentration, reduced acetate-to-propionic acid ratio, and elevated microbial protein production. From the perspective of rumen microbial community, BP increased the diversity and evenness of rumen microbial. The relative abundance of Actinobacteriota, Fibrobacterota, *Olsenella*, *Fibrobacter*, *Lachnospiraceae_NK3A20_group*, and *Succiniclasticum* was significantly higher in the BP group than in the CON group (*p* < 0.05). In terms of metabolism, BP exerted positive effects on indicators related to hepatocyte damage, muscle injury, lipid metabolism, protein metabolism, and endotoxin levels. For immune function, BP enhanced immune responses by reducing interleukin (IL)-1β and Tumor Necrosis Factor-α (TNF-α) levels (*p* < 0.05) and increasing immunoglobulin levels (*p* < 0.05), thereby improving overall immunity. Moreover, BP significantly enhanced the body’s antioxidant capacity by increasing the activities of antioxidant enzymes, such as superoxide dismutase (SOD) and glutathione peroxidase (GSH-Px) (*p* < 0.05). Correlation analysis revealed that major acute-phase protein, TNF-α, and IL-1β all exhibited significant negative correlations with certain differential microorganisms (*p* < 0.05). In conclusion, 0.5% BP supplementation in HCD improves goat production performance by optimizing rumen fermentation and microbial community, enhancing immunity and antioxidant capacity, and regulating metabolic function, providing a theoretical basis for BP application in ruminant production under high-concentrate feeding conditions.

## 1 Introduction

In modern ruminant production, high-concentrate diets (HCD) are widely used to improve production performance and shorten the fattening periods (1, 2). Nevertheless, long-term HCD feeding disrupts rumen microecological homeostasis, increases the risk of rumen acidosis, and triggers systemic inflammation as well as oxidative stress via the release of endotoxin. Ultimately impairing animal health and production efficiency (3, 4). In traditional livestock production, antibiotics such as monensin have been commonly used to enhance rumen fermentation and overall health of ruminants fed a high-grain diet (5, 6). However, with the prohibition of antibiotic, research has increasingly focused on exploring natural and green additives to mitigate the adverse effects of HCD.

Bee pollen (BP), a natural substance derived from pollen and bee secretions collected by honeybees, is widely recognized as a “high-value nutritional resource” (7). It is abundant in a variety of nutrients, including proteins, essential amino acids, polysaccharides, vitamins, minerals, trace elements, and diverse bioactive compounds. The biologically active components of BP, including flavonoids, polyphenols, and polysaccharides, exert a multiple of biological functions, such as growth promotion, anti-inflammatory and immunomodulatory activities, as well as potent antioxidant capacity (8–11). These inherent functional properties endow BP with substantial potential as an antibiotic alternative. As a natural and green feed additive, BP has been increasingly adopted in livestock and poultry production. For instance, Fengyan et al. (12) demonstrated that dietary BP supplementation optimized gastric structure, enhanced digestive and absorptive efficiency, thereby improving production performance of Wanxi White Goose. Attia et al. (13) reported that continuous or intermittent BP supplementation positively influenced antioxidant enzyme activities, immune function, body weight gain, and lymphoid organ development in broilers. In porcine studies, Wang and Li (14) concluded that BP facilitated nutrient absorption and intestinal tissue development in the small intestine of fattening pigs, stimulated the proliferation of small intestinal epithelial cells, and enhanced humoral immune function. These studies demonstrate the beneficial effects of BP as a natural feed additive on livestock and poultry.

In current intensive farming, fattening sheep or goat production are typically fed a high-concentrate total mixed ration (TMR), which poses a substantial challenge to rumen homeostasis and overall animal health. Although numerous studies have highlighted the beneficial effects of BP on production performance, immunity, and metabolism in ruminants (10, 15). However, previous studies have lacked an in-depth assessment of the effects of BP on rumen homeostasis and overall health, and the application potential of BP in HCD remains unclear. Therefore, the objective of the present study was to systematically evaluate the effects of BP supplementation on rumen fermentation characteristics, rumen microbiota, serum biochemistry, immune function, antioxidant indices and production performance in goats fed HCD. This study aims to innovatively elucidate the application potential of BP in HCD and provide a scientific theoretical basis for the use of BP in ruminant production.

## 2 Materials and methods

### 2.1 Experimental animals and design

The Animal Care and Use Committee of Sichuan Agricultural University approved this research (SCAUAC201408-3). Twenty male growing Jintang Black goats with similar body weights (33.36 ± 1.34 kg) were selected as experimental animals. A single-factor experimental design was adopted. The goats were randomly assigned to two treatment groups: the CON group (fed the basal diet) and the BP group (fed the basal diet + 0.5% broken BP, dry matter basis, added as a premix). Each group contained 10 replicates, with one goat per replicate. The total trial period was 48 days. First, a 10-day pre-feeding period was used to allow the goats to adapt to the diet. The formal trial period was 38 days, with a growth trial carried out from Day 1 to Day 30. On Day 31, the experimental goats were transferred to digestion cages, and a digestion trial was conducted from Day 31 to Day 38 (the diet remained unchanged, including a 2-day adaptation period and a 6-day sample collection period).

### 2.2 Animal management and experimental diet

The trial was conducted at the Ya’an Teaching and Research Base of Sichuan Agricultural University. The experimental diet (Table 1) was formulated according to the “Nutritional Requirements for Meat Sheep and Goats (NY/T816-2021)” (16). During the trial period, the goats were fed twice daily at 9:00 a.m. and 5:00 p.m. All goats had free access to the experimental diet, and sufficient feed was provided to ensure that approximately 20% of the feed remained leftover each day. The bee pollen used in the experiment was provided by Sichuan Fenghui Natural Technology Co., Ltd. (Sichuan, China) (contained: crude protein ≥20.0%, ether extract ≥5.0%, total sugars ≥30.0%, polysaccharide ≥8.0%, polypeptide ≥7.0%, flavonoids ≥2.0%, polyphenols ≥1.0%).

**Table 1:** Composition and nutrients content of experimental diets (air dry basis).

Item	Group^a^	
	CON	BP
Ingredients		
Corn	50.20	50.20
Soybean meal	10.00	10.00
DDGS	6.00	6.00
Alfalfa hay	20.00	20.00
Corn husk	10.00	10.00
CaCO_3_	0.80	0.80
NaCl	0.40	0.40
Premix^b^	0.55	0.55
NaHCO_3_	1.00	1.00
MgO	0.50	0.50
Antioxidant	0.05	0.05
Mixed bran	0.50	0.00
Bee pollen	0.00	0.50
Chemical composition		
Dry matter	88.37	88.37
Crude protein	14.50	14.50
Crude ash	6.70	6.70
Ether extract	3.22	3.22
Metabolizable energy	2.57	2.57
Neutral detergent fiber	47.09	47.09
Acid detergent fiber	11.73	11.73
Calcium	0.58	0.58
Phosphorus	0.41	0.41
Concentrate:roughage	80:20	80:20

a CON, Basic diet group; BP, basic diet + 0.5% bee pollen group.

b The premix provided the following per kg of diet: vitamin A 2200 IU; vitamin D 250 IU; vitamin E 20 IU; Fe 40 mg; Cu 10 mg; Zn 30 mg; Mn 40 mg; I 0.8 mg; Se 0.2 mg; Co 0.11 mg. Added via premix form.

### 2.3 Sample collection

#### 2.3.1 Collection of feed and feces sample

Representative samples of the experimental diet were collected using the quartering method. After thorough mixing, samples were sealed and stored for subsequent nutrient analysis.

During days 33–38 of the formal trial, total feces from each goat were collected twice daily (at approximately 09:00 and 17:00). The collected feces were weighed and recorded, and daily feces from each goat were pooled to form a composite sample. Composite samples were then air-dried and stored at −20 °C until analysis for nutrient digestibility.

#### 2.3.2 Collection of rumen sample

On day 30 of the formal trial, rumen contents were collected from all goats prior to the morning feeding. Briefly, goats were restrained and a stomach tube connected to a vacuum pump was inserted orally into the rumen. The tube position was adjusted as necessary to facilitate collection. The electric pump was then activated to extract the contents. One portion was aliquoted into sterile 10 mL centrifuge tubes, wrapped in aluminum foil, labeled, and stored at −80 °C for subsequent microbial analysis. Another portion of the contents was filtered through 4 layers of gauze. The resulting filtrate was aliquoted, and its pH was measured immediately, these aliquots were then stored at −20 °C for later analysis of rumen fermentation parameters.

#### 2.3.3 Collection of blood sample

On the morning of day 30 of the formal trial, blood samples (10 mL per goat) were collected from the jugular vein into vacuum tubes. After collection, the samples were kept at room temperature for 30 min to clot, followed by centrifugation at 3000×g for 15 min at 4 °C. The resulting serum was aliquoted into three portions and stored at −20 °C for until analysis for antioxidant, immune, and biochemical parameters.

### 2.4 Laboratory analysis

#### 2.4.1 Production performance

Throughout the 30-day formal trial, daily feed intake and feed refusals were recorded for each goat. Initial body weight (IBW; recorded on day 1) and final body weight (FBW; recorded on day 30) were measured. Average daily feed intake (ADFI), average daily gain (ADG), and feed-to-gain ratio (F/G) were subsequently calculated.

#### 2.4.2 Apparent digestibility of nutrients

The collected feed and fecal samples were oven-dried at 65 °C for 48 h, ground using a mill (ShengShun SS-1022, China), and passed through a 1 mm sieve. The contents of dry matter (DM), crude ash, calcium (Ca) and phosphorus (P) were determined according to AOAC method (17). Ether extract (EE) was analyzed by the Soxhlet extraction method (17). Crude protein (CP) content was determined using a Kjeldahl nitrogen analyzer (BUCHI K-355, Aptar, Switzerland) following the Kjeldahl method. Neutral detergent fiber (NDF) and acid detergent fiber (ADF) contents were analyzed with a fiber analyzer (ANKIM A2000i, Wellness, United States) according to Van Soest et al. (18). The apparent digestibility of nutrients was calculated using the following formula:


$$\text{Digestibility}(\%)=\frac{\begin{array}{l} \text{Quality of nutritional intake}(g)-\\ \text{Nutrient content in feces}(g) \end{array}}{\text{Quality of nutritional intake}(g)}\times 100\%$$

#### 2.4.3 Rumen fermentation parameters

The pH of rumen fluid was measured immediately with a portable pH meter (PHBJ-260, China). Microbial crude protein (MCP) concentration was determined by differential centrifugation (19), followed by quantification using a BCA protein assay kit. Ammonia nitrogen (NH_3_-N) content was analyzed via the phenol- hypochlorite spectrophotometry method (20). Volatile fatty acids (VFAs) content was determined by gas chromatograph-mass spectrometry (GC-MS, Agilent Technologies, Palo Alto, CA, United States) according to the method of Colin et al. (21).

#### 2.4.4 Blood parameters

Serum concentrations of immunoglobulins (IgM, IgA, IgG), interleukins (IL-1β, IL-6, IL-10), tumor necrosis factor-α (TNF-α), lipopolysaccharide (LPS) and histamine (HIS) were measured using enzyme-linked immunosorbent assay (ELISA) kits (Meimian Biotechnology, Yancheng, China) according to the manufacturer’s instructions.

The levels of oxidative stress markers, including malondialdehyde (MDA), glutathione peroxidase (GSH-Px), superoxide dismutase (SOD), protein carbonyl (PCO), and total antioxidant capacity (T-AOC), were determined in serum using respective micro assay kits (Solarbio, Beijing, China) following the provided protocols.

Serum biochemical parameters—alanine transaminase (ALT), aspartate transaminase (AST), creatinine (CREA), urea, alkaline phosphatase (ALP), lactate dehydrogenase (LDH), creatine kinase (CK), total protein (TP), albumin (ALB), total cholesterol (TC), triglycerides (TG), low-density lipoprotein cholesterol (LDL-C), and high-density lipoprotein cholesterol (HDL-C)—were analyzed with a fully automatic biochemical analyzer (Hitachi 3100, Hitachi, Tokyo, Japan).

#### 2.4.5 Rumen microbial composition

Rumen fluid (1 mL) was centrifuged at 12000×g for 10 min at 4 °C, and the resulting pellet was collected. Genomic DNA was extracted using the TIANamp Bacteria DNA Kit (Tianjin, China) following the CTAB method (22). DNA purity and concentration were assessed by 1% agarose gel electrophoresis and a NanoDrop 8000 spectrophotometer (Thermo Fisher Scientific, Brisbane, QLD, Australia).

The V3-V4 hypervariable region of the bacterial 16S rRNA gene was amplified with primers 515F (5′-GTGCCAGCMGCCGCGGTAA-3′) and 806R (5′-GGACTACHVGGGTWTCTAAT-3′) (23). Sequencing libraries were constructed using the NEBNext® Ultra II DNA Library Prep Kit (New England Biolabs Ltd., China) and paired-end sequenced (2 × 300 bp) on an Illumina MiSeq platform.

Raw reads were demultiplexed, merged using FLASH (v1.2.11), and quality-filtered to generate clean tags. Chimeric sequences were detected and removed by comparing clean tags against the SILVA (SSU rRNA database for high-quality ribosomal RNA gene sequences) database via USEARCH yielding effective tags. After denoising with the Divisive Amplicon Denoising Algorithm 2 (DADA2) plugin in Quantitative Insights into Microbial Ecology 2 (QIIME2), amplicon sequence variants (ASVs) were obtained and taxonomically classified against the SILVA database.

Alpha diversity (Chao1, Dominance, Observed_otus, Pielou_e, Shannon, Simpson) was calculated in QIIME2, and species accumulation curves were generated. Beta diversity was assessed via principal coordinate analysis (PCoA) based on weighted UniFrac distances. Permutational multivariate analysis of variance (PERMANOVA) and analysis of similarity (ANOSIM) in R were used to evaluate intergroup differences in microbial structure. Taxonomic composition at the phylum and genus levels was visualized using bar plots, and differential relative abundance were analyzed using the Mann–Whitney *U* test with false discovery rate (FDR) correction for multiple testing.

### 2.5 Correlation between rumen microbiota and blood parameters

Nonparametric Spearman’s rank correlation analysis was conducted in SPSS 27.0 to evaluate the correlation between microorganisms exhibiting intergroup differences in relative abundance and blood parameters (immune and antioxidant indices). Correlations were corrected for multiple comparisons using the FDR. The resulting correlations were visualized as a heatmap generated using Origin (2021).

### 2.6 Statistical analysis

Statistically analyzed using SPSS 27.0. The independent-sample *t*-test was used when the data conformed to the normal distribution. The Mann–Whitney *U* test was used when the data did not meet the normal distribution. *p*-values were corrected by FDR multiple testing. Results were considered statistically significant at *p* < 0.05 and highly significant at *p* < 0.01.

## 3 Results

### 3.1 Production performance

The effects of BP supplementation on the production performance of goats are shown in Table 2. Compared with the CON group, the average daily gain (ADG) (*p* = 0.016) and average daily feed intake (ADFI) (*p* = 0.042) of the BP group were significantly increased, with respective increments of 36.55 and 15%. Meanwhile, the feed-to-gain ratio (F/G) was significantly decreased in the BP group (*p* = 0.034).

**Table 2:** Effects of supplementing bee pollen to high-concentrate diets on the production performance of goats.

Item	Diet^a^		SEM	*p*-value
	CON	BP		
Initial body weight, IBW (kg)	33.4	33.32	1.57	0.516
Final body weight (day 30) (kg)	37.75	39.25	2.77	0.113
Average daily gain, ADG (kg/d)	0.145	0.198	0.01	0.016
Average daily feed intake, ADFI (kg/d)	1.13	1.30	0.05	0.042
Feed-to-gain ratio, F/G	7.79	6.56	0.81	0.034

a CON, Basic diet group; BP, basic diet + 0.5% bee pollen group.

### 3.2 Apparent digestibility of nutrients

The effects of BP supplementation on nutrient apparent digestibility in goats are shown in Table 3. Compared with the CON group, the apparent digestibility of DM, CP, and NDF in the BP group was significantly increased (*p* < 0.05). These results suggest that dietary BP supplementation enhanced the digestibility of key nutrients in goats fed HCD.

**Table 3:** Effects of supplementing bee pollen to high-concentrate diets on the nutrient digestibility of goats.

Item	Diet^a^		SEM	*p*-value
	CON	BP		
Dry matter, DM	65.81	72.69	1.56	0.022
Crude protein, CP	67.35	73.73	1.86	0.035
Acid detergent fiber, ADF	48.18	49.55	1.02	0.134
Neutral detergent fiber, NDF	63.88	72.49	0.92	0.041
Ether extract, EE	75.99	81.27	3.23	0.087

a CON, Basic diet group; BP, basic diet + 0.5% bee pollen group.

### 3.3 Rumen fermentation parameters

The effects of BP supplementation on rumen fermentation parameters are shown in Table 4. No significant differences were observed between groups in rumen fluid pH, the concentrations of acetate, propionate, butyrate, isobutyrate, isovalerate, and NH₃-N (*p* > 0.05). However, compared with the CON group, the BP group showed significantly higher concentration of valeric acid (*p* = 0.042) and MCP (*p* = 0.045), along with a significantly lower acetate-to-propionate ratio (A:P) (*p* = 0.031).

**Table 4:** Effects of supplementing bee pollen to high-concentrate diets on rumen fermentation parameters of goats.

Item	Diet^a^		SEM	*p*-value
	CON	BP		
pH value	6.66	6.82	0.08	0.356
TVFA (mmol/L)	148.14	155.64	12.99	0.782
Acetate (mmol/L)	85.69	79.78	6.17	0.646
Propionate (mmol/L)	46.49	60.20	6.15	0.276
Butyrate (mmol/L)	15.53	15.15	1.72	0.115
Isobutyrate (mmol/L)	0.12	0.11	0.01	0.286
Isovalerate (mmol/L)	0.15	0.11	0.01	0.060
Valeric (mmol/L)	0.16	0.29	0.03	0.042
A:P^b^	2.28	1.52	0.16	0.031
MCP (μg/L)	22.91	30.72	1.98	0.045
NH_3_-N (mg/dL)	47.28	44.35	0.95	0.125

a CON, Basic diet group; BP, basic diet + 0.5% bee pollen group; TVFA, total volatile fatty acids.

b A:P, Acetate to propionate ratio; MCP, microbial protein.

### 3.4 Blood parameters

The effects of BP supplementation on serum biochemical indices are shown in Table 5. Compared with the CON group, the BP group exhibited significantly lower serum levels of AST, urea, CK, TC, TG, LPS, and HIS (*p* < 0.05), along with significantly higher serum levels of ALP and ALB (*p* < 0.05). No significant intergroup differences were observed in the serum levels of ALT, CREA, LDH, TP, LDL-C, and HDL-C (*p* > 0.05).

**Table 5:** Effects of supplementing bee pollen to high-concentrate diets on blood biochemical parameters of goats.

Item	Diet^a^		SEM	*p*-value
	CON	BP		
Alanine aminotransferase, ALT (U/L)	24.04	21.76	0.91	0.222
Aspartate aminotransferase, AST (U/L)	80.98	72.18	2.06	0.028
UREA (mmol/L)	11.79	8.82	0.63	0.013
Creatinine, CREA (μmol/L)	91.97	78.82	6.76	0.351
Alkaline phosphatase, ALP (U/L)	326.1	734.1	105.25	0.049
Lactate dehydrogenase, LDH (U/L)	316.43	310.18	13.27	0.821
Creatine kinase, CK (U/L)	239.48	126.14	31.03	0.046
Total protein, TP (g/L)	70.19	67.63	0.92	0.170
Albumin, ALB (g/L)	31.38	33.89	0.59	0.035
Total cholesterol, TC (mmol/L)	1.97	1.70	0.07	0.042
Triglyceride, TG(mmol/L)	0.44	0.34	0.03	0.039
Low-density lipoprotein cholesterol, LDL-C (mmol/L)	0.74	0.80	0.07	0.655
High-density lipoprotein cholesterol, HDL-C (mmol/L)	1.46	1.48	0.06	0.851
Lipopolysaccharide, Lps (ng/L)	435.92	383.75	10.68	0.009
Histamine, HIS (ng/mL)	14.13	12.62	0.38	0.041

a CON, Basic diet group; BP, basic diet + 0.5% bee pollen group.

The effects of BP supplementation on serum immune indices in goats are shown in Table 6. Compared with the CON group, the BP group showed significantly lower serum levels of IL-1β, TNF-α, and MAP (*p* < 0.05), and significantly higher serum levels of IgM, IgG (*p* < 0.05), as well as IgA (*p* < 0.01).

**Table 6:** Effects of supplementing bee pollen to high-concentrate diets on serum immune parameters of goats.

Item	Diet^a^		SEM	*p*-value
	CON	BP		
Interleukin-1β, IL-1β (pg/mL)	83.32	65.78	3.79	0.016
Interleukin-6, IL-6 (pg/mL)	89.56	94.96	3.82	0.494
Interleukin-10, IL-10 (pg/mL)	25.68	26.51	1.54	0.796
Tumor necrosis factor-α, TNF-α (pg/mL)	150.12	133.55	4.14	0.042
Immunoglobulin M, IgM (μg/mL)	1329.48	1540.07	52.90	0.047
Immunoglobulin A, IgA (μg/mL)	134.64	181.98	8.18	0.001
Immunoglobulin G, IgG (mg/mL)	5.29	6.17	0.21	0.030
Major acute phase protein, MAP (mg/L)	161.74	133.86	5.94	0.015
Heat shock protein 70, HSP-70 (pg/mL)	447.71	392.78	16.37	0.094

a CON, Basic diet group; BP, basic diet + 0.5% bee pollen group.

The effects of BP supplementation on serum antioxidant indices are shown in Table 7. Compared with the CON group, the BP group exhibited significantly higher serum levels of T-AOC (*p* = 0.033), GSH-Px (*p* = 0.046), and SOD (*p* = 0.009).

**Table 7:** Effects of supplementing bee pollen to high-concentrate diets on serum antioxidants of goats.

Item	Diet^a^		SEM	*p*-value
	CON	BP		
Malondialdehyde, MDA (nmol/mL)	1.02	0.89	0.07	0.347
Total antioxidant capacity, T-AOC (μmol/mL)	0.11	0.15	0.01	0.033
Glutathione peroxidase, GSH-Px (U/mL)	27.98	31.64	0.93	0.046
Superoxide dismutase, SOD (U/mL)	1.62	2.08	0.11	0.009

a CON, Basic diet group; BP, basic diet + 0.5% bee pollen group.

### 3.5 Analysis of rumen microbial 16S rRNA sequencing

#### 3.5.1 Amplicon sequence variant (ASV) analysis

**Figure 1:**
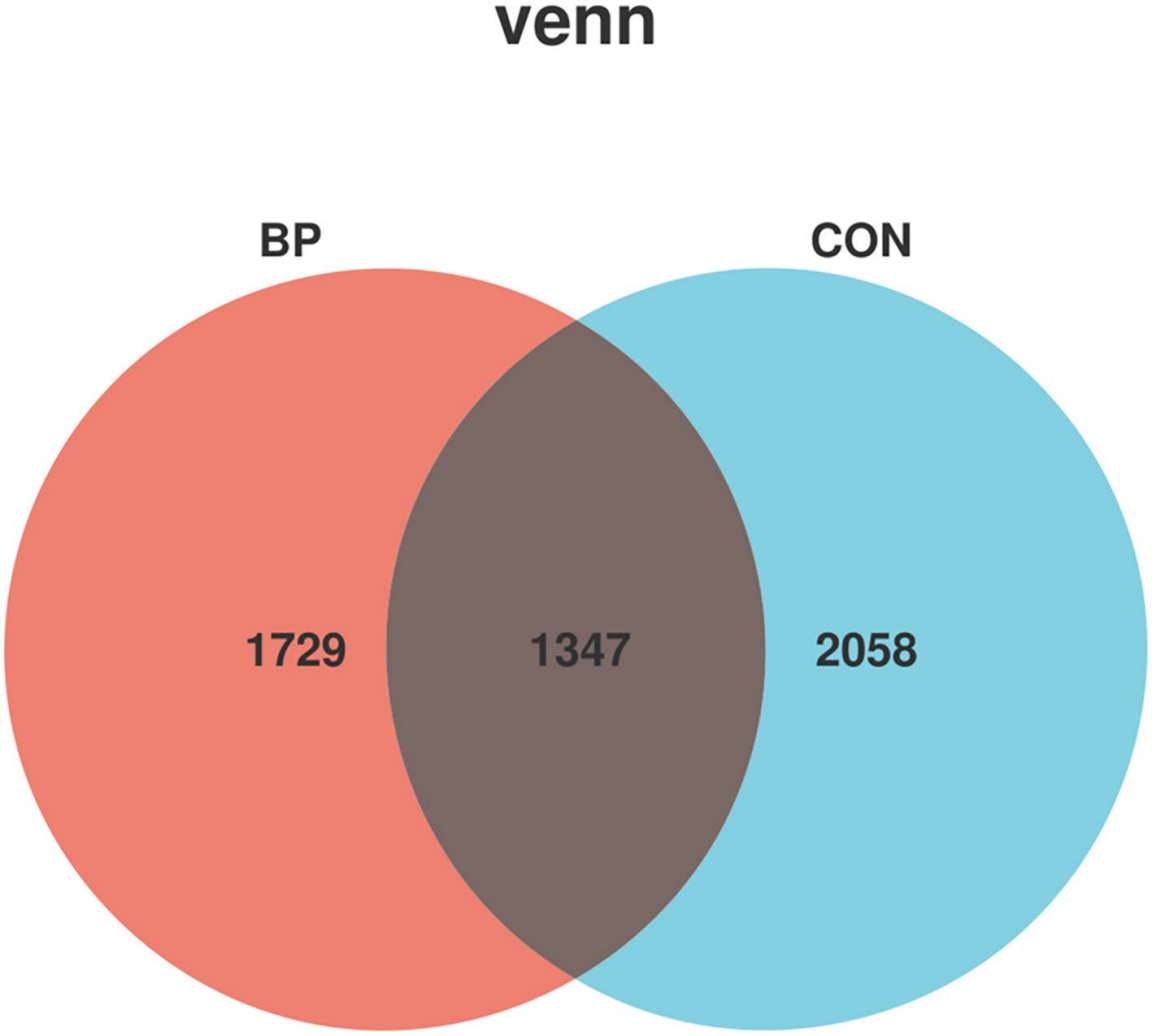
Venn diagram of ASVs in rumen microbiota of goats in the CON group and BP group.

Divisive Amplicon Denoising Algorithm 2 (DADA2) was employed to denoise the sequencing data, with each non-chimeric, high-quality sequence defined as an ASV. Following denoising, a total of 1,347 ASVs were shared between the two groups (Figure 1), while unique and common ASVs across groups were further analyzed.

#### 3.5.2 Alpha diversity analysis

The effects of BP supplementation on the alpha diversity of the rumen microbiota are shown in Table 8. Compared with the CON group, the BP group exhibited significantly higher Pielou-e (*p* = 0.039), Shannon (*p* = 0.046), and Simpson (*p* = 0.024) indices, along with a significantly lower Dominance index (*p* = 0.024).

**Table 8:** Effects of supplementing bee pollen to high-concentrate diets on alpha diversity of rumen microbiota in goats.

Item	Diet^a^		SEM	*p*-value
	CON	BP		
Chao1	720.80	794.00	33.97	0.279
Dominance	0.09	0.03	0.01	0.024
Observed_otus	762.30	690.80	31.61	0.272
Pielou_e	0.62	0.71	0.02	0.039
Shannon	5.91	6.76	0.21	0.046
Simpson	0.91	0.97	0.01	0.024

a CON, basic diet group; BP, basic diet + 0.5% bee pollen group.

#### 3.5.3 Beta diversity analysis

**Figure 2:**
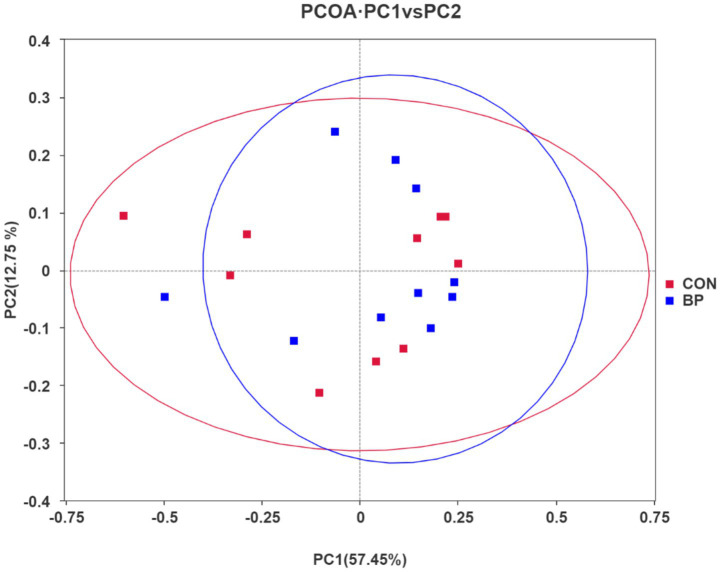
Principal coordinate (PCoA) analysis plot of rumen microbial communities in goats in the CON group and BP group (PC1 accounts for 57.45%, PC2 accounts for 25%). PCoA based on weighted UniFrac distances revealed distinct clustering patterns between groups. The CON group exhibits greater sample dispersion, whereas the BP group forms a tighter cluster, indicating smaller individual differences among samples in the BP group.

Principal coordinate analysis (PCoA) based on weighted UniFrac distances revealed distinct clustering patterns between groups (Figure 2; PC1 accounts for 57.45%, PC2 account for 25%). PERMANOVA analysis showed that the rumen microbial community structure was significantly different between the two groups (*R*^2^ = 0.216, *p* = 0.002), and ANOSIM analysis further confirmed the significant intergroup difference (*R* = 0.324, *p* = 0.003). Samples from the CON group displayed greater dispersion, reflecting higher inter-individual variation in rumen microbial composition. In contrast, samples from the BP group formed a more tightly clustered profile, indicating that BP supplementation reduced individual variability and promoted greater stability in the rumen microbiota.

#### 3.5.4 Microbiota composition in rumen

**Figure 3:**
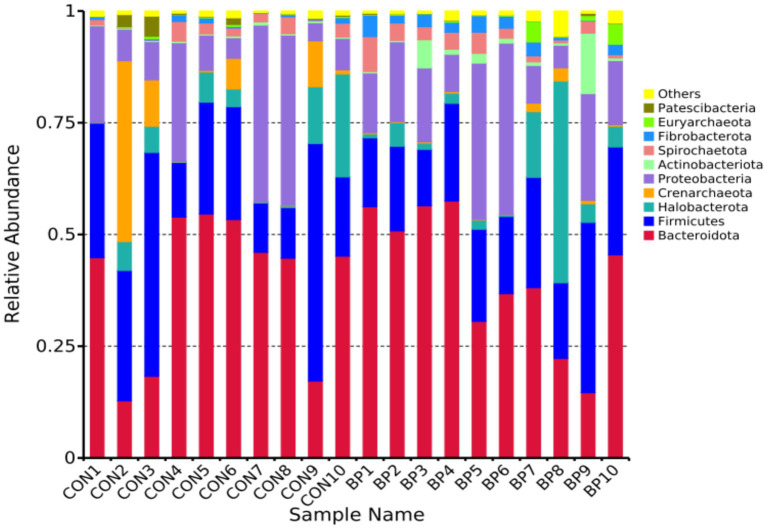
Relative abundance of dominant rumen bacteria at the phylum level (top 10) in goats in the CON group and BP group.

The composition of rumen bacteria at the phylum and genus levels was analyzed to evaluate the effect of BP supplementation. At the phylum level (Figure 3), Bacteroidota and Firmicutes collectively accounted for over 90% of the total bacterial abundance in both groups, with no significant intergroup difference in their relative abundance (*p* > 0.05). In contrast, the relative abundances of Actinobacteriota and Fibrobacterota were significantly higher in the BP group compared with the CON group (*p* < 0.01; Table 9).

**Table 9:** Differences in the relative abundance of dominant rumen microorganisms between the CON group and BP group at the phylum and genus levels (%).

Item	Diet^a^		SEM	*p*-value
	CON	BP		
Phylum				
Actinobacteriota	0.34	2.68	0.70	0.004
Fibrobacterota	0.61	2.42	0.31	0.003
Genus				
*Olsenella*	0.23	2.55	0.71	0.002
*Fibrobacter*	0.61	2.42	0.31	0.003
*Lachnospiraceae_NK3A20_group*	0.18	0.70	0.10	0.001
*Succiniclasticum*	0.42	0.86	0.12	0.043

a CON, basic diet group; BP, Basic diet + 0.5% bee pollen group.

**Figure 4:**
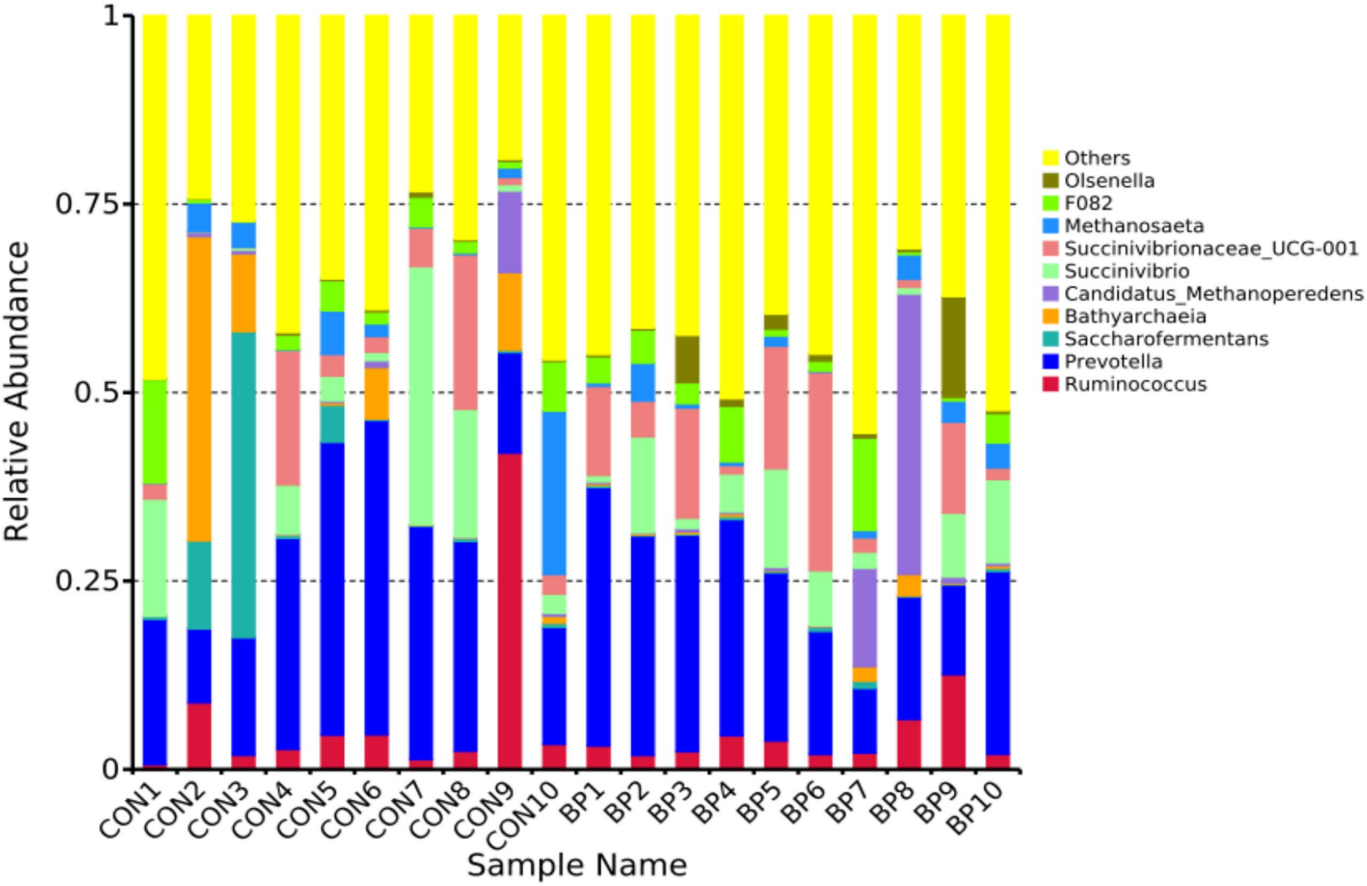
Relative abundance of dominant rumen bacteria at the genus level (top 10) in goats in the CON group and BP group.

At the genus level (Figure 4), *Ruminococcus* and *Prevotella* predominated in both groups. Compared with the CON group, the BP group showed significantly higher relative abundances of *Olsenella*, *Fibrobacter*, *Lachnospiraceae_NK3A20_group*, and *Succiniclasticum* (*p* < 0.05; Table 9). These findings suggest that BP supplementation modulates rumen microbial composition, particularly by enriching beneficial genera associated with fiber degradation and propionate synthesis.

#### 3.5.5 Correlation between rumen microbiota and blood parameters

**Figure 5:**
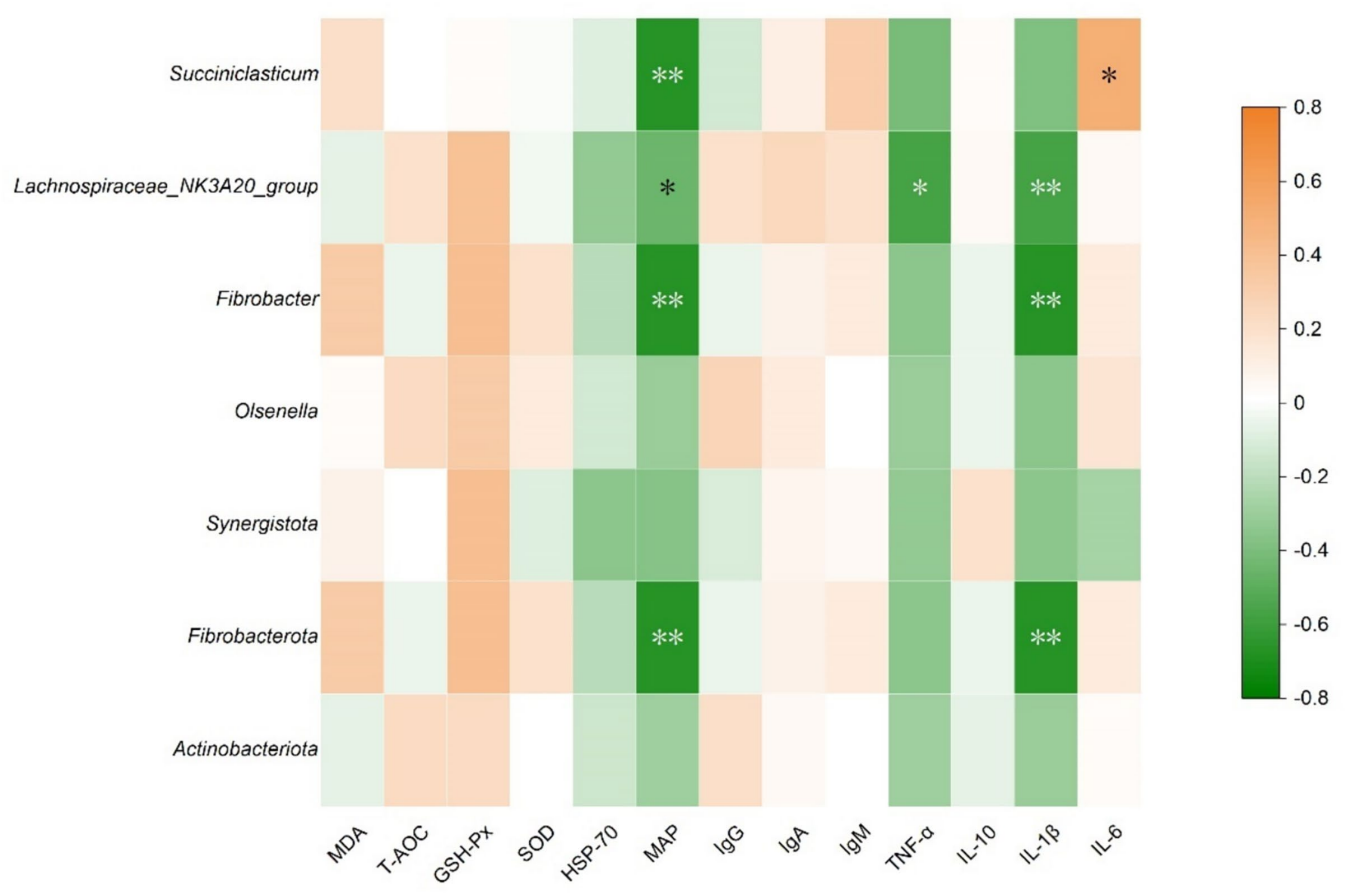
Heatmap of the correlation between rumen differential microorganisms and serum immune/antioxidant indices (*p < 0.05; **p < 0.01).

Correlations between differentially abundant rumen microorganisms and serum immune/antioxidant indices are presented in Figure 5. The relative abundances of Fibrobacterota and *Fibrobacter* showed significant negative correlations with IL-1β (*R* = −0.562, *p* = 0.010) and MAP (*R* = −0.668, *p* < 0.01). Similarly, the relative abundance of *Lachnospiraceae_NK3A20_group* was negatively correlated with IL-1β (*R* = −0.534, *p* = 0.015) and TNF-α (*R* = −0.468, *p* = 0.038). In contrast, *Succiniclasticum* abundance was positively correlated with IL-6 (*R* = 0.514, *p* = 0.020). These results imply that BP supplementation may modulate host immune responses through shifts in rumen microbial composition, thereby supporting health of goats fed HCD.

## 4 Discussion

In the present study, supplementation of BP into the HCD significantly increased the ADG and reduced the F/G in goats, indicating a positive effect of BP on production performance. Which aligns with previous reports in calves (15). The results also indicate that BP significantly increased the ADFI of goats. This may be due to the bioactive substances contained in BP, which are involved in regulating the physiological functions of animals. Previous studies found that flavonoid compounds can reduce the gene expression of bitter taste receptors (TAS2R) in the rumen and duodenal epithelium of beef cattle (24, 25). Activation of TAS2R promotes the release of anorexigenic hormones such as cholecystokinin (CCK) and peptide YY (PYY) (25), which inhibit feed intake. Therefore, BP may reduce TAS2R gene expression through flavonoid compounds, thereby decreasing the release of anorexigenic hormones, which in turn increases dry matter intake and promotes growth.

The rumen fermentation parameters were examined to further explain the effects of BP on rumen homeostasis. Rumen pH is a pivotal indicator of rumen health (26). In the current study, the rumen pH of the BP group was slightly higher than that of the CON group, albeit with no significant statistical difference. This phenomenon may be attributed to the buffering capacity of polysaccharides, organic acids, and minerals present in BP, which can neutralize excess lactic acid and acetic acid in the rumen, potentially exerting a preventive effect against rumen acidosis (27, 28). However, the underlying mechanism of this action requires to be further investigated. The results of this study demonstrated that BP increased the concentration of MCP in goat rumen fluid. MCP serves as an important protein source for ruminants, and its synthesis is closely associated with rumen fermentation and microbial activity (29). Bee pollen supplementation not only maintains an optimal rumen environment, but also directly provides microorganisms with the “nitrogen skeleton” required for synthesizing bacterial protein, thereby meeting the substrate demands for microbial protein synthesis (30). This indicates that BP promotes ammonia nitrogen conversion and facilitates MCP synthesis. VFA can reflect the rumen fermentation pattern (31, 32). The A:P ratio reflects the energy metabolism status of ruminants. A lower A:P ratio is beneficial for improving energy conversion efficiency, promoting body weight gain, and reducing methane emissions and energy loss (33). In this study, the A:P ratio in the BP group was significantly lower than that in the CON group, which may be related to the changes in rumen microbial composition. Under the influence of BP, the relative abundance of *Olsenella* was significantly increased. *Olsenella* can convert lactate into pyruvate via lactate dehydrogenase, and pyruvate can be further metabolized into propionate (34), thereby increasing the proportion of propionate and decreasing the A:P ratio. Additionally, the relative abundance of *Lachnospiraceae_NK3A20_group* was also significantly elevated in this study. Previous studies have reported that the abundance of *Lachnospiraceae_NK3A20_group* is positively correlated with propionate concentration (35), this group can ferment glucose to produce lactic acid, which is then converted into propionate by other microorganisms (36), further contributing to the increase in propionate concentration. BP exerts a positive effect on the rumen bacterial community, particularly on the genus *Fibrobacter*. *Fibrobacter* is one of the primary cellulolytic bacteria in the rumen, capable of secreting cellulase to break down cellulose into fermentable carbohydrates (35). Furthermore, *Fibrobacter* and *Lachnospiraceae_NK3A20_group* may synergistically participate in fiber digestion, jointly promoting improved fiber digestibility. *Succiniclasticum* is a genus capable of converting succinate into propionate. *Fibrobacter* can produce succinate through unique metabolic pathways (37), and the increased relative abundance of *Fibrobacter* and *Succiniclasticum* may promote the conversion of succinate to propionate, thereby enhancing propionate concentration and facilitating energy conversion. Moreover, BP supplementation significantly increased the diversity and evenness of rumen microbiota, which is beneficial for maintaining the stability of the rumen microbial community and improving the adaptability of microorganisms to environmental changes. This result may be associated with the antibacterial activity of BP (38). Various microorganisms in the rumen exhibit symbiotic, antagonistic, and competitive relationships. It is plausible that the bioactive compounds in BP promote the growth of beneficial competitive microorganisms by inhibiting the proliferation of harmful bacteria. For example, flavonoids (e.g., quercetin) can reduce the abundance of protozoa and methanogens while competitively promoting the growth of rumen bacteria (39–41).

Bee pollen has a significant impact on the metabolism of goats. Serum levels of TC and TG were significantly lower in the BP group, suggesting that BP may regulate lipid metabolism in goats (42–44). Its potential lipid-lowering mechanisms are attributed to unsaturated fatty acids (such as linolenic acid) in BP, which promote cholesterol metabolism and excretion (45, 46). It is also possible that BP contains phytosterols, which have a structure similar to cholesterol and can competitively inhibit intestinal cholesterol absorption (47, 48). AST is an important serum marker associated with hepatocyte integrity; the significantly lower serum AST level in the BP group indicates an improvement in serum indicators related to hepatocyte damage, which is consistent with previous studies in broilers (49). We propose that this protective effect on hepatocyte-related serum indicators may be due to the antioxidant bioactive substances (e.g., flavonoids, polyphenols, and polysaccharides) in BP, which can alleviate hepatic oxidative stress and reduce hepatocyte damage (50). ALB and UREA levels reflect the balance between protein synthesis and amino acid metabolism. The significantly higher ALB and lower UREA levels in the BP group suggest an improvement in serum indicators related to protein metabolism. The abundant high-quality amino acids in BP provide ample raw materials for protein synthesis (51) and the balanced amino acid composition in BP may enables efficient absorption and utilization, reducing nitrogenous waste generated by protein breakdown (52). Additionally, BP protects liver function, and the liver is the main organ for protein synthesis; thus, the improvement of liver function is beneficial for protein synthesis (53).

Previous studies have confirmed that long-term feeding of HCD lowers rumen pH and increases the permeability of the rumen epithelium, leading to the translocation of LPS into the bloodstream. Once in the circulatory system, LPS binds to Toll-like receptor 4 (TLR4) on immune cells (54), activating the nuclear factor-κB (NF-κB) signaling pathway, promoting the release of pro-inflammatory cytokines, and inducing systemic inflammation. This imposes stress on the body’s immune system. In the present study, we found that BP supplementation significantly decreased the serum levels of pro-inflammatory cytokines (IL-1β and TNF-α) and endotoxins (LPS and HIS), while markedly increasing the serum levels of immunoglobulins (IgM, IgA and IgG). These findings indicate that BP is beneficial for alleviating inflammation and enhancing immune function, which is consistent with previous reports (55–57). Based on the results of this study, we propose that these effects of BP may be attributed to two mechanisms. First, BP stabilizes rumen pH to reduce the release of LPS and HIS, thereby indirectly inhibiting the activation of inflammatory signaling pathways (58). Second, the bioactive compounds in BP exert immunostimulatory effects. Specifically, components such as polysaccharides and polyphenols in BP may promote the development of immune organs, including the spleen and lymphoid tissues, enhance the activity of immune cells (13, 59–62), and further facilitate the secretion of immunoglobulins. These mechanisms require further verification.

BP contains natural antioxidants such as polysaccharides, phenolic compounds, vitamins, and flavonoids, and can activate and enhance the body’s own antioxidant enzyme defense system (38, 63–65). In this trial, BP supplementation significantly increased the serum levels of T-AOC, GSH-Px, and SOD. SOD and GSH-Px are key enzymes in combating oxidative stress, which can effectively scavenge free radicals and exert primary antioxidant effects (66). SOD can convert superoxide anions into hydrogen peroxide, and GSH-Px can convert hydrogen peroxide into water, thereby scavenging ROS. The increase in the serum levels of SOD and GSH-Px in the BP group indicates that BP enhances the activity of antioxidant enzymes, which is beneficial for scavenging ROS and alleviating oxidative stress. T-AOC comprehensively reflects the combined antioxidant capacity of all antioxidant substances within the organism. Based on these indicators, we found that BP exerts a positive effect on the antioxidant capacity of goats.

The rumen microbiota is closely related to the health and production performance of ruminants (67). Correlation analysis revealed that the relative abundance of Fibrobacterota, *Fibrobacter*, and *Lachnospiraceae_NK3A20_group* was significantly negatively correlated with the levels of pro-inflammatory cytokines (IL-1β and TNF-*α*) and the MAP. This suggests a potential link between these microorganisms and immune regulation in goats. We propose potential explanations for this correlation: The active proliferation of *Fibrobacter* may indirectly inhibit other VFA-producing bacteria by competing for substrates, thereby reducing VFA accumulation in the rumen (68), maintaining rumen homeostasis, and indirectly decreasing endotoxin release; *Lachnospiraceae_NK3A20_group* is involved in and promotes the synthesis of short-chain fatty acid (SCFA) (69, 70), and SCFAs may promote the proliferation of rumen epithelial cells, enhance rumen epithelial tight junctions, and reduce epithelial permeability (71, 72), thereby decreasing endotoxin entry into the bloodstream and reducing pro-inflammatory cytokine release. These results suggest that the differential microorganisms enriched by BP may be involved in the immune regulation of goats fed HCD, but the direct causal relationship requires further verification.

**Figure 6:**
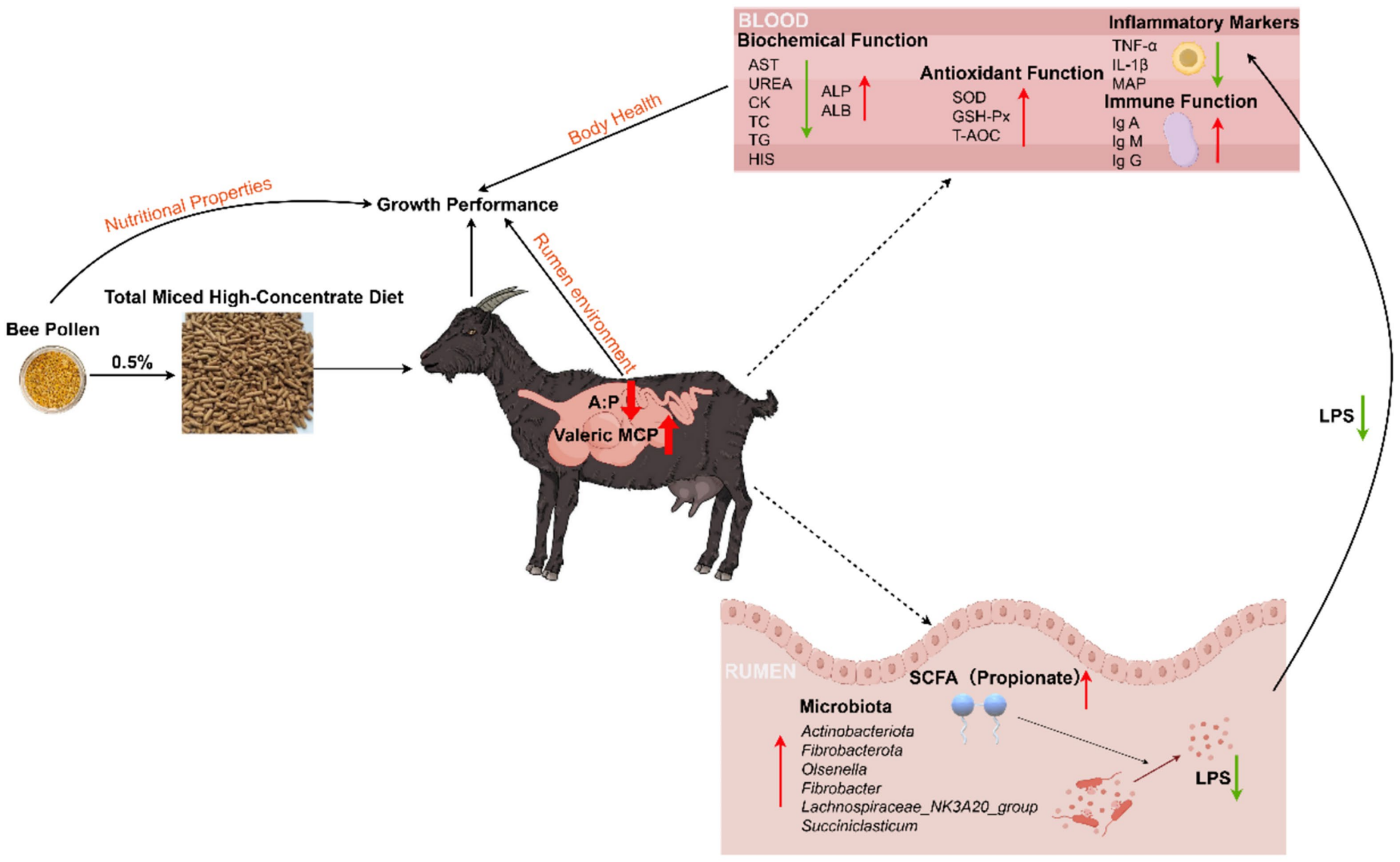
Supplementing 0.5% bee pollen to a high concentrate diet enhances goat performance in three aspects: improving nutritional characteristics, optimizing rumen environment, and maintaining overall health. (A:P = Acetate to Propionate ratio; MCP = Microbial protein; SCFA = Short Chain Fatty Acids; LPS = Lipopolysaccharide; TNF-α = Tumor Necrosis Factor-α; IL = Interleukin; MAP = Major Acute-phase Protein; Ig = Immunoglobulin; SOD = Superoxide Dismutase; GSH-Px = Glutathione Peroxidase; T-AOC = Total Antioxidant Capacity; AST = Aspartate aminotransferase; CK = Creatine Kinase; TC = Total Cholesterol; TG = Triglyceride; HIS = Histamine; ALP = Alkaline Phosphatase; ALB = Albumin).

Based on the above results, the effects of BP on the production performance and overall health of goats are evident across multiple aspects (Figure 6). Specifically, BP enhances rumen fermentation patterns and optimizes rumen microbial composition, thereby promoting the proliferation of beneficial rumen bacteria. In terms of overall health regulation, BP primarily exerts its effects by suppressing the levels of pro-inflammatory factors, increasing the concentrations of immunoglobulins, and enhancing the body’s antioxidant capacity. Collectively, improvements in rumen function and body health ultimately translate to a positive impact on the production performance of goats.

## 5 Conclusion

Supplementation of HCD with 0.5% BP significantly enhanced rumen fermentation in goats, leading to increased valeric and microbial protein, and reduced the acetic-to-propionic ratio, thereby improving rumen fermentation efficiency. Additionally, BP supplementation modulated the rumen microbial composition by promoting the proliferation of beneficial bacteria. It also enhanced goats’ immunity and antioxidant capacity while positively influencing metabolism. Furthermore, dietary BP improved nutrient digestibility and growth performance. These findings demonstrate that BP supplementation is a beneficial strategy for supporting ruminant health and production under HCD feeding conditions.

## Data Availability

The raw sequencing data has been deposited in the Sequence Read Archive (SRA) at the NCBI, https://www.ncbi.nlm.nih.gov/, PRJNA1452465.
